# Identification of Potential Signatures and Their Functions for Acute Lymphoblastic Leukemia: A Study Based on the Cancer Genome Atlas

**DOI:** 10.3389/fgene.2021.656042

**Published:** 2021-07-06

**Authors:** Weimin Wang, Chunhui Lyu, Fei Wang, Congcong Wang, Feifei Wu, Xue Li, Silin Gan

**Affiliations:** Department of Hematology, The First Affiliated Hospital of Zhengzhou University, Zhengzhou, China

**Keywords:** acute lymphoblastic leukemia, long non-coding RNAs, functional enrichment analysis, competing endogenous RNAs, hub genes interaction

## Abstract

**Objective:**

Acute lymphoblastic leukemia (ALL) is a malignant disease most commonly diagnosed in adolescents and young adults. This study aimed to explore potential signatures and their functions for ALL.

**Methods:**

Differentially expressed mRNAs (DEmRNAs) and differentially expressed long non-coding RNAs (DElncRNAs) were identified for ALL from The Cancer Genome Atlas (TCGA) and normal control from Genotype-Tissue Expression (GTEx). DElncRNA–microRNA (miRNA) and miRNA–DEmRNA pairs were predicted using online databases. Then, a competing endogenous RNA (ceRNA) network was constructed. Functional enrichment analysis of DEmRNAs in the ceRNA network was performed. Protein–protein interaction (PPI) network was then constructed. Hub genes were identified. DElncRNAs in the ceRNA network were validated using Real-time qPCR.

**Results:**

A total of 2,903 up- and 3,228 downregulated mRNAs and 469 up- and 286 downregulated lncRNAs were identified for ALL. A ceRNA network was constructed for ALL, consisting of 845 lncRNA-miRNA and 395 miRNA–mRNA pairs. These DEmRNAs in the ceRNA network were mainly enriched in ALL-related biological processes and pathways. Ten hub genes were identified, including SMAD3, SMAD7, SMAD5, ZFYVE9, FKBP1A, FZD6, FZD7, LRP6, WNT1, and SFRP1. According to Real-time qPCR, eight lncRNAs including ATP11A-AS1, ITPK1-AS1, ANO1-AS2, CRNDE, MALAT1, CACNA1C-IT3, PWRN1, and WT1-AS were significantly upregulated in ALL bone marrow samples compared to normal samples.

**Conclusion:**

Our results showed the lncRNA expression profiles and constructed ceRNA network in ALL. Furthermore, eight lncRNAs including ATP11A-AS1, ITPK1-AS1, ANO1-AS2, CRNDE, MALAT1, CACNA1C-IT3, PWRN1, and WT1-AS were identified. These results could provide a novel insight into the study of ALL.

## Introduction

Acute lymphoblastic leukemia (ALL) is a malignant disease most commonly diagnosed in adolescents and young adults, especially in patients younger than 15 years. Despite significant improvements in the management of ALL, the long-term survival rate of ALL patients, especially adult patients, remains low (Jabbour et al., 2018; Richard-Carpentier et al., 2019). Therefore, it is of importance to understand the pathogenesis of ALL and identify novel diagnostic biomarkers and therapeutic targets for ALL.

LncRNA is a type of RNA longer than 200 nucleotides. Dysregulated lncRNA as tumor suppressor genes or oncogenes plays a key role in a variety of biological processes, such as cell proliferation, apoptosis, migration, and invasion. Increasing studies are focusing on the role and mechanism of lncRNA in the occurrence and development of ALL (Trimarchi et al., 2014; Arthur et al., 2017). For instance, lncRNA CASC15 could regulate SOX4 expression in RUNX1-translocated leukemia (Fernando et al., 2017). LncRNA HOTAIR is closely associated with acute leukemia patients’ poor prognosis (Zhang et al., 2016). LncRNA HOXA-AS2 induces glucocorticoid resistance by promoting ALL cell proliferation and inhibiting apoptosis (Zhao et al., 2019). Despite the fact that many studies have shown the diagnostic and prognostic values of lncRNAs in ALL, it is still required to further understand their regulatory mechanism. It has been widely accepted that lncRNAs indirectly regulate gene expression through targeted miRNAs (about 20 nucleotides) at the transcriptional or post-transcriptional level. Many miRNAs have been found to play a functional regulatory role in the development of ALL, such as miRNA-126 (Nucera et al., 2016), miRNA-155 (El-Khazragy et al., 2019), and miR-141-3p (Zhou et al., 2019). Yet, the regulatory interactions between lncRNAs and miRNAs in ALL require to be clarified.

The development of transcriptome analysis and RNA sequencing technology is increasing the possibility of identifying lncRNAs that may be involved in the pathogenesis of ALL. Moreover, further studies on the function of abnormally expressed lncRNAs may help understand the pathogenesis of ALL and provide important insights for the treatment of ALL. In this study, we comprehensively analyzed DElncRNAs and DEmRNAs in bone marrow samples of ALL. A ceRNA network was constructed for ALL on the basis of DElncRNA–miRNA and miRNA–DEmRNA pairs. DEmRNAs in the ceRNA network were significantly associated with ALL-related biological processes and pathways. Among DElncRNAs in the ceRNA network, eight lncRNAs including ATP11A-AS1, ITPK1-AS1, ANO1-AS2, CRNDE, MALAT1, CACNA1C-IT3, PWRN1, and WT1-AS were validated by Real-time qPCR, which could become potential diagnostic and therapeutic targets of ALL.

## Materials and Methods

### ALL Data Acquisition and Differential Expression Analysis

LncRNA and mRNA RNA-seq data of 494 bone marrows with ALL (hematopoietic and reticuloendothelial systems) were retrieved from TCGA repository^1^, which were derived from the IlluminaHiSeq RNA-Seq platform. All the data from three phases together, including 12 cases of phase 1, 468 cases of phase 2, and 14 cases of phase 3 were enrolled in the study. There were 321 (64.98%) males, 172 (34.82%) females, and 1 unknown (0.02%). The age distribution of the ALL group is as follows: 403 cases of 0–14 years old and 91 cases of ≥ 14 years old. All data of normal tissue samples were obtained from 407 whole blood in the Genotype-Tissue Expression (GTEx) database^2^. There were 265 (65.11%) males and 142 (34.89%) females in the control group. The age distribution of the control group is as follows: 34 cases of 20–29 years old, 34 cases of 30–39 years old, 72 cases of 40–49 years old, 130 cases of 50–59 years old, 132 cases of 60–69 years old, and 5 cases of 70–79 years old. Complete description of the multiple ethnicity groups, the biospecimen procurement methods, and sample fixation was provided in the GTEx official annotation. Differential expression analyses between ALL samples and normal samples were carried out using the EdgeR package in R (Robinson et al., 2010). The obtained *p*-values were corrected by false discovery rate (FDR). mRNAs and lncRNAs with adjusted *p* < 0.05 and | log 2fold change (FC)| ≥ 2 were considered as DEmRNAs and DElncRNAs. Volcano plots and heatmaps were generated using the ggplot2 and packages in R, respectively.

### ceRNA Network Construction

After identification of DElncRNAs and DEmRNAs, lncRNA–miRNA pairs were predicted by miRcode^3^ that provides > 10,000 lncRNAs (Jeggari et al., 2012). Then, miRNAs that targeted DEmRNAs were predicted using TargetScan^4^ (Agarwal et al., 2015), miRDB^5^ (Wang, 2008), and miRTarBase database^6^, which provides an experimentally validated microRNA–target interactions database (Huang et al., 2020). After integration of DElncRNA–miRNA and miRNA–DEmRNA, a ceRNA network was constructed and visualized using the Cytoscape software (version 3.5.1) (Shannon et al., 2003).

### Functional Enrichment Analyses of DEmRNAs in the ceRNA Network

Gene Ontology (GO) analysis of DEmRNAs in the ceRNA network was carried out using Database for Annotation, Visualization, and Integrated Discovery (DAVID) (Dennis et al., 2003), including biological process (BP), cellular component (CC), and molecular function (MF). Moreover, Kyoto Encyclopedia of Genes and Genomes (KEGG) was analyzed using the clusterProfiler in R (Yu et al., 2012). Furthermore, the KEGG results were visualized using the Cytoscape plug-in ClueGO. *p* < 0.05 was set as the cutoff value.

### PPI Network

The interactions between proteins were predicted using the Search Tool for the Retrieval of Interacting Genes (STRING) database^7^ (minimum required interaction score > 0.4) (Szklarczyk et al., 2019). Furthermore, PPI networks were embodied using the Cytoscape v3.5.0 software. In addition, we used Molecular Complex Detection (MCODE) plugin to identify the hub genes in the PPI network. The criteria were set as follows: MCODE scores > 3 and number of nodes > 4. The top 10 hub genes were identified using the ranking method of degree.

### Real-Time qPCR

Bone marrow samples were isolated from 25 ALL patients and 15 healthy participants and red blood cells were removed. Total RNA was extracted from bone marrow samples and then was stored at −80℃. Extracted samples were lysed using 1 ml of Trizol and placed for 5 min on ice. RNA concentration and purity were determined using a NanoDrop UV spectrophotometer. Then, RNA was reverse transcribed into cDNA. Primer sequences of ATP11A-AS1, ITPK1-AS1, ANO1-AS2, CRNDE, MALAT1, CACNA1C-IT3, PWRN1, and WT1-AS were designed and synthesized by Shanghai Shengong Biological Engineering Co., Ltd. (Shanghai, China). The primer sequences are listed in Table 1. PCR amplification had the following conditions: 95℃ for 3 min; 40 PCR cycle reactions (95℃ for 20 s; 60℃ for 30 s). GAPDH was used as a control. Relative expression levels of lncRNAs were calculated using 2^–ΔΔCT^ method. Differences between the two groups were analyzed using Student’s *t*-test. *p*-value < 0.05 was considered statistically significant.

**TABLE 1:** Primer sequence information for Real-time qPCR.

Gene symbol	Primer sequence (5′–3′)
Human GAPDH	5′-CGGAGTCAACGGATTTGGTCGTAT-3′ (forward) 5′-AGCCTTCTCCATGGTGGTGAAGAC-3′ (reverse)
Human ANO1-AS2	5′-CCGGAACAAGAACCTCGCTC-3′ (forward) 5′-GGTCCTCGCCTACCATCCAA-3′ (reverse)
Human PWRN1	5′-ACATTCGAAACCCAGGTGCC-3′ (forward) 5′-GGAAGTGGATGCTGACGCTC-3′ (reverse)
Human MALAT1	5′-GGTTCAGAAGGTCTGAAGCTC-3′ (forward) 5′-CCCAGAAGTGTTTACACTGCT-3′ (reverse)
Human CACNA1C-IT3	5′-GCCAGGACCAAGACACCAAGAC-3′ (forward) 5′-TTGGGCAGGGCTCGGTTCC-3′ (reverse)
Human ITPK1-AS1	5′-AATCCTGTGCGCTGTCATCC-3′ (forward) 5′-GATTGCTCTTGGCTGTGCCT-3′ (reverse)
Human ATP11A-AS2	5′-ACAGTCCCTTCCCTTACGCT-3′ (forward) 5′-TGAACGCTGCACTTGTGGAC-3′ (reverse)
Human CRNDE	5′-GAGGACGTGCTGGGGCT-3′ (forward)
	5′-CTGAGTCCATGTCCCGAATC-3′(reverse)
Human WT1-AS	5′-GCCTCTCTGTCCTCTTCTTTGT-3′ (forward)
	5′-GCTGTGAGTCCTGGTGCTTAG-3′ (reverse)

## Results

### Identification of DElncRNAs and DEmRNAs for ALL

**FIGURE 1:**
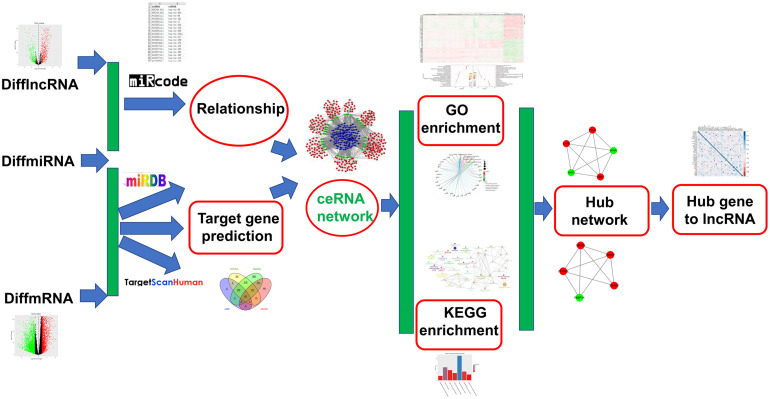
The workflow of this study.

**FIGURE 2:**
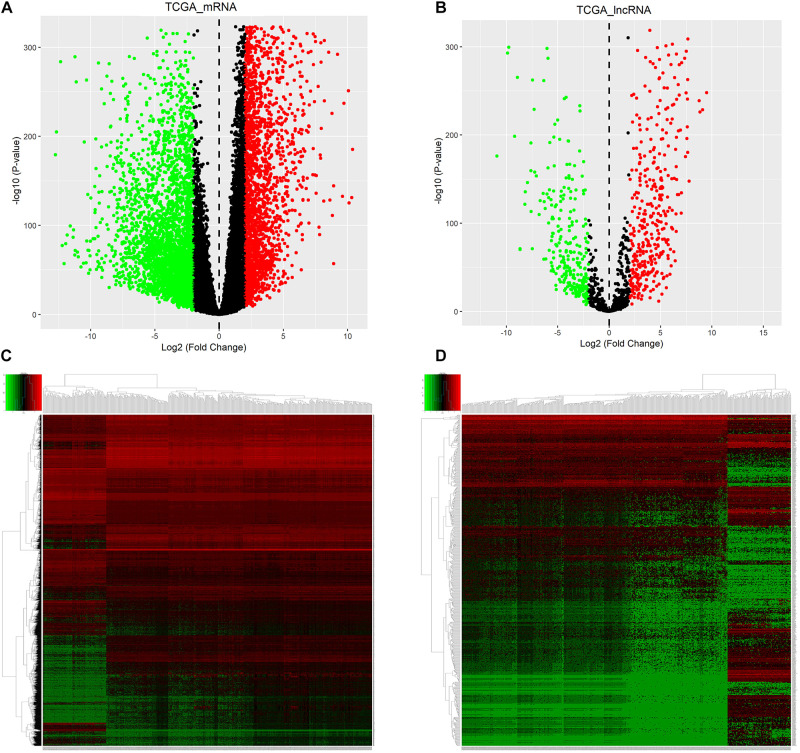
Identification of DEmRNAs and DElncRNAs for ALL. Volcano plot showing DEmRNAs **(A)** and DElncRNAs **(B)** for ALL. As shown in heatmaps, the differences in expression patterns of DEmRNAs **(C)** and DElncRNAs **(D)** between ALL bone marrow samples and normal samples. Red represents upregulation and green represents downregulation. DEmRNAs: differentially expressed mRNAs; DElncRNAs: Differentially expressed lncRNAs; ALL, Acute lymphoblastic leukemia.

The workflow of this study is shown in Figure 1. According to adjusted *p*-value < 0.05 and | log2 fold change (FC)| ≥ 2, 2903 up− and 3228 downregulated mRNAs were identified for ALL, as shown in the volcano plot (Figure 2A and Supplementary Material 1). Furthermore, there were 469 up− and 286 downregulated lncRNAs for ALL (Figure 2B and Supplementary Material 2). Heatmaps depicted the differences in expression patterns of all DEmRNAs (Figure 2C) and DElncRNAs (Figure 2D) between ALL bone marrow samples and normal samples.

### Construction of ceRNA Network for ALL

**FIGURE 3:**
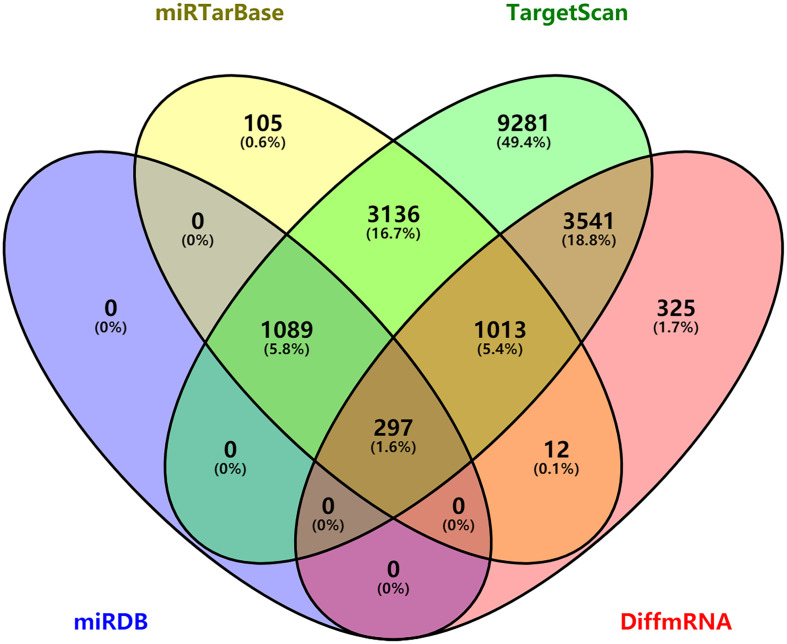
Venn diagram showing 297 differentially expressed mRNAs targeted by miRNAs *via* intersection of prediction results of TargetScan, miRDB, and miRTarBase database.

**FIGURE 4:**
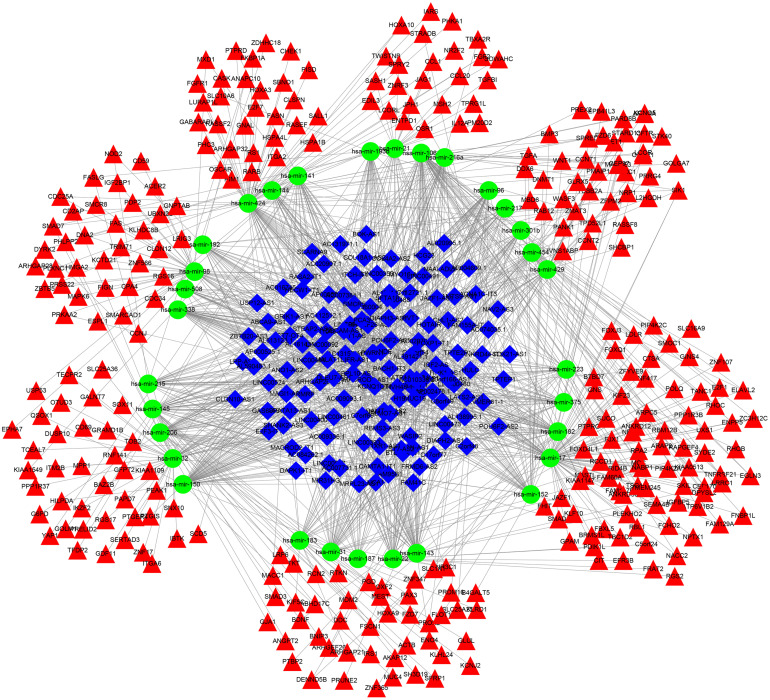
A ceRNA network construction for acute lymphoblastic leukemia. Blue rhombus represents lncRNAs; green circle represents miRNAs and red triangle represents mRNAs.

The miRNAs that targeted DEmRNAs were predicted using TargetScan, miRDB, and miRTarBase databases. After integration of prediction results from the three databases, 297 DEmRNAs were intersected and identified for the construction of ceRNA network (Figure 3). Furthermore, DElncRNA–miRNA relationships were predicted using miRcode database. By comprehensively analyzing DElncRNA–miRNA and miRNA–DEmRNA pairs, a ceRNA network was constructed for ALL (Figure 4). There were 845 lncRNA–miRNA pairs (Supplementary Material 3) and 395 miRNA–mRNA pairs (Supplementary Material 4) in the ceRNA network.

### Functional Enrichment Analysis of DEmRNAs in the ceRNA Network

**FIGURE 5:**
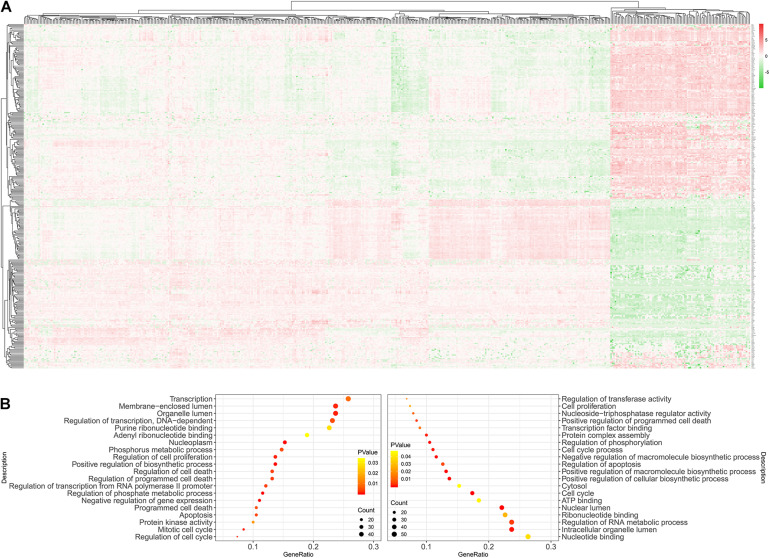
GO enrichment analysis of DEmRNAs in the ceRNA network. **(A)** Heatmaps showing differences in the expression patterns of all DEmRNAs in the ceRNA network between ALL bone marrow samples and normal samples. Red stands for upregulation and green stands for downregulation. **(B)** The top 40 GO enrichment analysis results including biological process, cellular component, and molecular function. DEmRNAs: Differentially expressed mRNAs.

**FIGURE 6:**
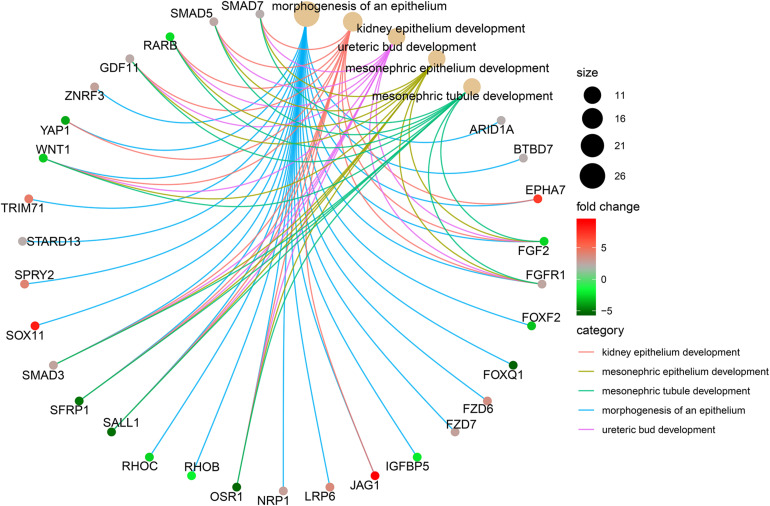
The top five biological processes enriched by DEmRNAs in the ceRNA network. DEmRNAs: Differentially expressed mRNAs.

**FIGURE 7:**
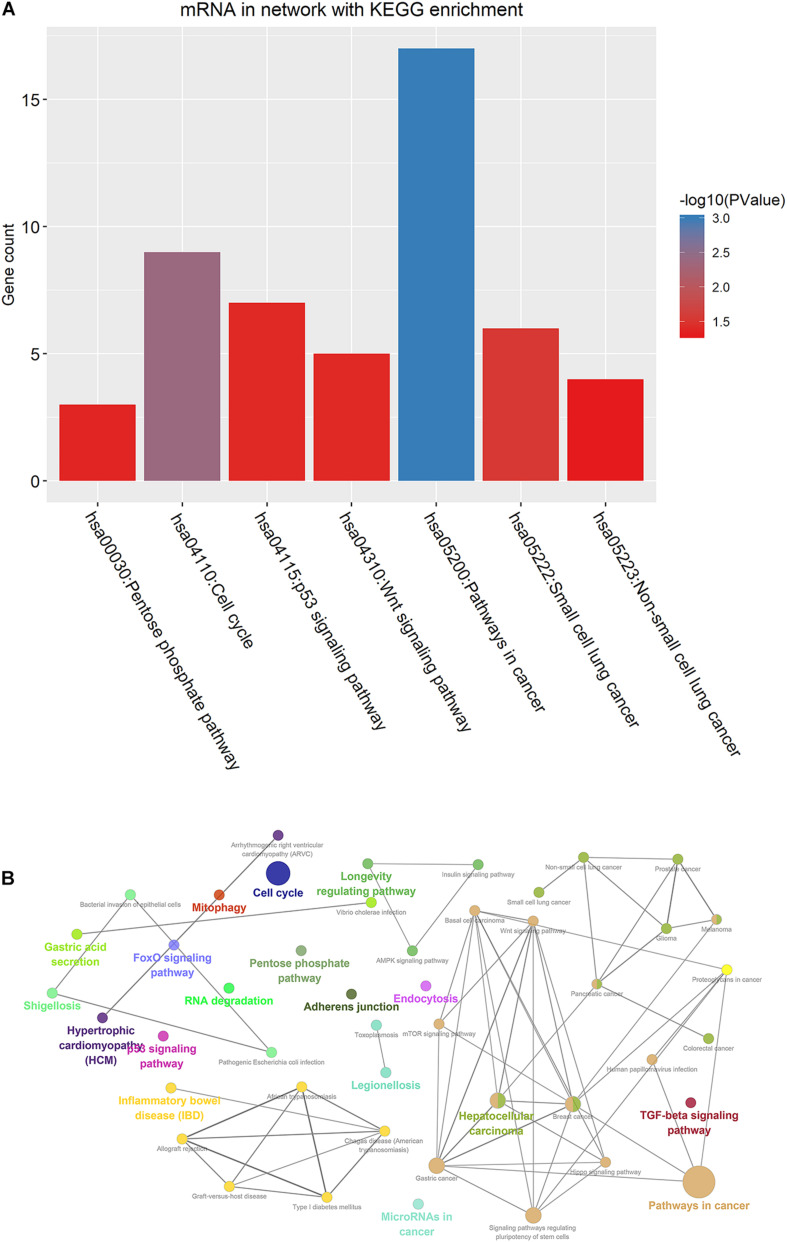
KEGG pathway enrichment analysis of DEmRNAs in the ceRNA network. **(A)** Seven enriched KEGG pathways. **(B)** Visualization of KEGG enrichment analysis results. DEmRNAs: Differentially expressed mRNAs.

As depicted in heatmaps, there were obvious differences in the expression patterns of all DEmRNAs in the ceRNA network between ALL bone marrow samples and normal samples (Figure 5A). Bubble diagrams showed the top 40 GO enrichment analysis results enriched by DEmRNAs in the ceRNA network (Figure 5B). We found that these mRNAs were mainly enriched in ALL-related biological processes such as transcription, programmed cell death, apoptosis, cell cycle, proliferation, and so on. Figure 6 depicted the relationships between DEmRNAs and enriched biological processes including morphogenesis of an epithelium, kidney epithelium development, ureteric bud development, mesonephric epithelium development, and mesonephric tubule development. As for KEGG pathway enrichment analysis results, these DEmRNAs were mainly enriched in pathways in cancer, cell cycle, small cell lung cancer, p53 signaling pathway, Wnt signaling pathway, pentose phosphate pathway, and non-small cell lung cancer (Figures 7A,B).

### Identification of Hub Genes in the PPI Network

**FIGURE 8:**
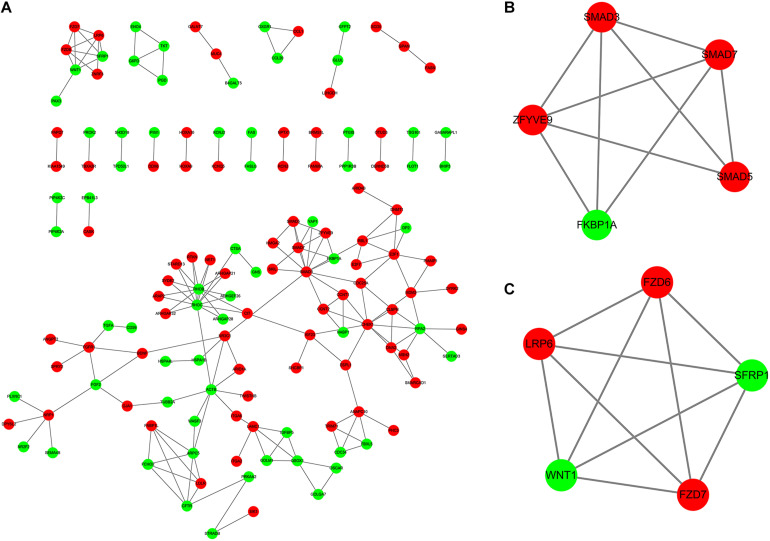
Identification of hub genes in the PPI network. **(A)** Construction of PPI network based on the DEmRNAs in the ceRNA network. **(B,C)** Two PPI subnetworks for ALL. Red represents upregulation and green represents downregulation. DEmRNAs: Differentially expressed mRNAs.

The DEmRNAs in the ceRNA network were imported into STRING database. Then, a PPI network was constructed for ALL (Figure 8A). Two PPI subnetworks were then constructed (Figures 8B,C). Ten hub genes were identified for ALL, including SMAD3, SMAD7, SMAD5, ZFYVE9, FKBP1A, FZD6, FZD7, LRP6, WNT1, and SFRP1.

### Correlation Between Hub Genes and DElncRNAs

**FIGURE 9:**
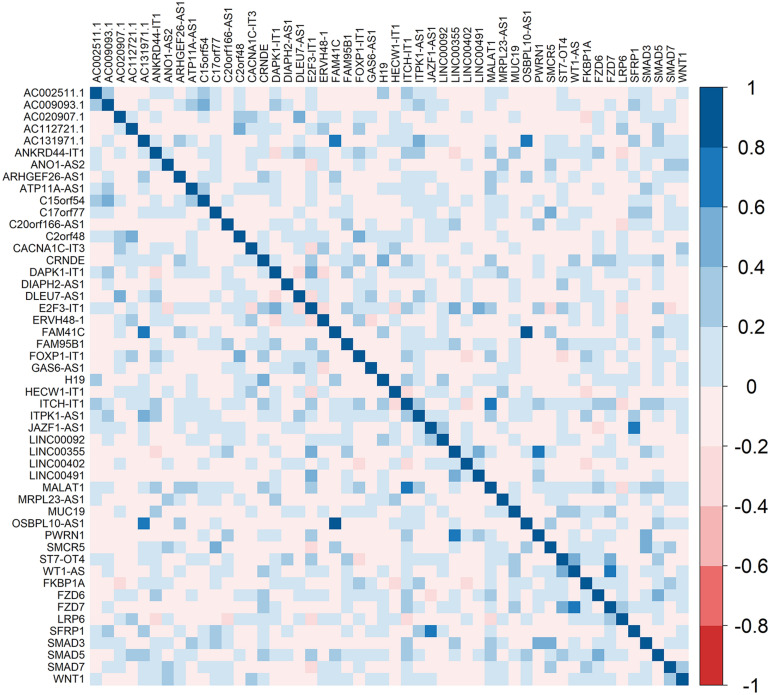
Heatmaps showing the correlation between hub genes and DElncRNAs. DEmRNAs: Differentially expressed mRNAs. The right bar indicates the color legend of Pearson correlation values.

Correlation analysis between hub genes and DElncRNAs was performed by corrplot package. The significant correlations between DElncRNAs and hub genes are shown in Figure 9 and Supplementary Material 5. There was strong correlation between WT1-AS and FZD7 (*r* = 0.751907203; *p* < 0.0001). Furthermore, PWRN1 and SMAD3 were significantly correlated (*r* = 0.521493415 and *p* = 4.32E−08).

### Validation of Eight lncRNAs in Bone Marrow of ALL

**FIGURE 10:**
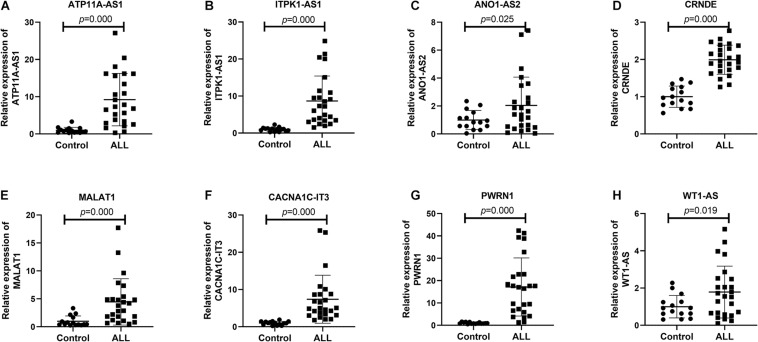
Validation of eight lncRNAs in ALL bone marrow samples using Real-time qPCR. **(A)** ATP11A-AS1; **(B)** ITPK1-AS1; **(C)** ANO1-AS2; **(D)** CRNDE; **(E)** MALAT1; **(F)** CACNA1C-IT3; **(G)** PWRN1; and **(H)** WT1-AS. Control: *n* = 15; ALL: *n* = 25.

Among all DElncRNAs in the ceRNA network, the most significant difference between 10 lncRNAs (AC009093.1, C17orf77, ATP11A-AS1, ITPK1-AS1, ANO1-AS2, ITCH-IT1, CRNDE, MALAT1, CACNA1C-IT3, and PWRN1) in the ceRNA network and WT1-AS (which was closely related to hub gene FZD7) was selected for verification. However, as the primers of AC009093.1, C17orf77, and ITCH-IT1 for RQ-PCR were not ideal, the remaining eight lncRNAs were validated. As Figure 10 shows, these eight lncRNAs were significantly upregulated in ALL bone marrow samples (*n* = 25) compared to normal samples (*n* = 15) by Real-time qPCR.

## Discussion

In this study, we constructed a ceRNA network for ALL based on DElncRNA–miRNA and miRNA–DEmRNA relationships. Among all DElncRNAs in the ceRNA network, eight lncRNAs were validated in ALL bone marrow samples using Real-time qPCR. These lncRNAs might become potential biomarkers for ALL.

To explore potential functions of DEmRNAs in the ceRNA network, we performed functional enrichment analysis. We found that these mRNAs were mainly enriched in ALL-related biological processes such as transcription (Gocho and Yang, 2019), programmed cell death (Hass et al., 2016), apoptosis, cell cycle (Jing et al., 2018), and proliferation (Sun et al., 2019). The DEmRNAs in these biological processes could modulate the development of ALL. Furthermore, these DEmRNAs were significantly associated with pathways in cancer, cell cycle, p53 signaling pathway, Wnt signaling pathway, and pentose phosphate pathway. It has been widely accepted that the p53 signaling pathway is a promising drug target in ALL (Trino et al., 2016). In particular, alterations of the tumor suppressor gene TP53 were frequently found in pediatric ALL (Demir et al., 2020). As for the Wnt signaling pathway, it was significantly correlated with the pathogenesis of ALL (Montano et al., 2018). Recent findings reported that inhibiting Wnt/β catenin could reverse multidrug resistance in children ALL (Fu et al., 2019). Moreover, the pathway is regulated by many factors. For example, miR-181a-5p could promote ALL cell proliferation *via* targeting the Wnt pathway (Lyu et al., 2017). Our results indicated that the DEmRNAs in the ceRNA network could be involved in the pathogenesis of ALL.

We constructed a PPI network for B-ALL on the basis of DEmRNAs in the ceRNA network. Ten hub genes were identified for ALL, including SMAD3, SMAD7, SMAD5, ZFYVE9, FKBP1A, FZD6, FZD7, LRP6, WNT1, and SFRP1. Among them, the loss of the Smad3 protein has been identified as a key feature of acute T-cell lymphoblastic leukemia (Wolfraim et al., 2004). Smad7 is a promising therapeutic target for B-cell ALL (Guo et al., 2018). Furthermore, microRNA-181a might regulate its expression for pediatric ALL (Nabhan et al., 2017). Wnt signaling pathway can enhance hematopoietic cell proliferation (Doubravska et al., 2008). It could mediate growth and prognosis of B-cell progenitor ALL, which could be a potential treatment strategy in ALL (Khan et al., 2007; Mochmann et al., 2011). In the pathway, FZD6, FZD7, LRP6, and WNT1 were marker proteins. LRP6 has been reported to be a candidate tumor suppressor gene in pre-B ALL (Montpetit et al., 2004). Furthermore, low expression of SFRP1 was significantly associated with clinical outcomes of patients with Philadelphia-positive ALL (Martin et al., 2008).

Consistently with differential expression analysis results, eight lncRNAs including ATP11A-AS1, ITPK1-AS1, ANO1-AS2, CRNDE, MALAT1, CACNA1C-IT3, PWRN1, and WT1-AS were significantly upregulated in ALL bone marrow, indicating that these abnormally expressed lncRNAs could be involved in the development of ALL. Among them, CRNDE was upregulated in the bone marrow of B-cell precursor acute lymphoblastic leukemia (BCP-ALL) patients and BCP-ALL cell lines (NALM-6 and RS4;11). Functionally, CRNDE upregulated CREB expression by suppressing miR-345-5p, thus promoting cell proliferation and reducing cell apoptosis in BCP-ALL (Wang W. et al., 2020). A large amount of research has reported that aberrantly expressed MALAT1 was involved in a variety of cancers, such as breast cancer metastasis (Kim et al., 2018), colon cancer (Wu et al., 2018), and non-small cell lung cancer (Li et al., 2018). Abnormally expressed is in significant association with poor prognosis in childhood ALL (Pouyanrad et al., 2019). Furthermore, miR-125b in combination with miR-99a and/or miR-100 could inhibit the expression of MALAT1 in vincristine-resistant children ALL cells (Moqadam et al., 2013). PWRN1 was significantly underexpressed in gastric cancer tissues and cells (Chen et al., 2018). Overexpressed PWRN1 could inhibit the proliferation and metastasis of gastric cancer cells and tumor growth. Furthermore, PWRN1 may regulate miR-425-5p expression by acting as its sponge in gastric cancer cells. ITPK1-AS1 expression could predict gastric cancer patients’ survival (Hu et al., 2019). WT1-AS has been characterized as a tumor-suppressive lncRNA in several cancers including cervical squamous cell carcinoma (Zhang et al., 2019), gastric cancer (Du et al., 2016), papillary thyroid carcinoma (Le et al., 2020), non-small cell lung cancer cell (Jiang et al., 2020), and hepatocellular carcinoma (Lv et al., 2015). Besides, WT1-AS can regulate WT1 on oxidative stress injury and apoptosis of neurons in Alzheimer’s disease *via* inhibition of the miR-375/SIX4 axis (Wang Q. et al., 2020). However, other lncRNAs have not been reported yet. According to our results, these lncRNAs deserve more research on ALL.

However, there are several limitations in this study. First, since there was no normal control of ALL in TCGA database, data of 407 whole blood in the GTEx database were obtained as control. Given that ALL primarily affects younger individuals, the age distribution of the control group is not ideally matched with the ALL from TCGA database, which may cause confounding. Second, the sample size of this study is small, and larger clinical samples should be used to verify these lncRNAs. In addition, this study lacks functional experiments. In future research, we will further the function and clinical value of these lncRNAs in ALL.

## Conclusion

In our study, a ceRNA network was constructed for ALL. Among all DElncRNAs in the ceRNA network, eight lncRNAs were validated in ALL bone marrow samples using Real-time qPCR, which might provide a novel insight into the further study of ALL.

## Data Availability Statement

The original contributions presented in the study are included in the article/Supplementary Material, further inquiries can be directed to the corresponding author/s.

## Ethics Statement

The studies involving human participants were reviewed and approved by the Institutional Research Ethics Committee of the First Affiliated Hospital of Zhengzhou University (2019-KY-194). The study was conducted in accordance with the Declaration of Helsinki. The patients/participants provided their written informed consent to participate in this study.

## Author Contributions

WW designed the project, proposed the research concept, and wrote the manuscript. WW, CL, and FWa constructed the bioinformatic analysis, constructed the graphic images and data charts, and performed the statistical processing. CW, FWu, XL, and SG performed the experiments. All authors read and approved the manuscript and agreed to be accountable for all aspects of the research in ensuring that the accuracy and integrity of any part of the work are appropriately investigated and resolved.

## Conflict of Interest

The authors declare that the research was conducted in the absence of any commercial or financial relationships that could be construed as a potential conflict of interest.

## Footnotes

1 https://portal.gdc.cancer.gov/

2 https://gtexportal.org/home/datasets

3 http://www.mircode.org/

4 http://www.targetscan.org/

5 http://www.mirdb.org/

6 http://mirtarbase.mbc.nctu.edu.tw/

7 http://string-db.org/

## Supplementary Material

Supplementary Material 1 Differentially expressed mRNAs.

Supplementary Material 2 Differentially expressed long non-coding RNAs.

Supplementary Material 3 845 lncRNA–miRNA pairs in the ceRNA network.

Supplementary Material 4 395 miRNA–mRNA pairs in the ceRNA network.

Supplementary Material 5 The specific *p*-values of correlation analysis between hub genes and DElncRNAs.

## Abbreviations

ALL: acute lymphoblastic leukemia
DEmRNAs: differentially expressed mRNAs
DElncRNAs: differentially expressed long non-coding RNAs
TCGA: The Cancer Genome Atlas
GTEx: Genotype-Tissue Expression
ceRNA: competing endogenous RNA
PPI: protein–protein interaction
FC: fold change
GO: Gene Ontology
BP: biological process
CC: cellular component
MF: molecular function
KEGG: Kyoto Encyclopedia of Genes and Genomes.
